# Neutrophil Extracellular Traps, Angiogenesis and Cancer

**DOI:** 10.3390/biomedicines10020431

**Published:** 2022-02-12

**Authors:** Remo Poto, Leonardo Cristinziano, Luca Modestino, Amato de Paulis, Gianni Marone, Stefania Loffredo, Maria Rosaria Galdiero, Gilda Varricchi

**Affiliations:** 1 Department of Translational Medical Sciences, University of Naples Federico II, 80131 Naples, Italy; remo.poto@gmail.com (R.P.); l.cristinziano@gmail.com (L.C.); modestinoluca@gmail.com (L.M.); depaulis@unina.it (A.d.P.); marone@unina.it (G.M.); stefanialoffredo@hotmail.com (S.L.); mrgaldiero@libero.it (M.R.G.); 2 Center for Basic and Clinical Immunology Research (CISI), University of Naples Federico II, 80131 Naples, Italy; 3 World Allergy Organization (WAO) Center of Excellence, 80131 Naples, Italy; 4 Institute of Experimental Endocrinology and Oncology (IEOS), National Research Council, 80131 Naples, Italy

**Keywords:** angiogenesis, angiopoietin, cancer, inflammation, neutrophil, neutrophil extracellular traps

## Abstract

Human neutrophils, the most abundant circulating leukocytes, are fundamental components of the host response against different pathogens. Until a few years ago, neutrophils received limited attention in cancer immunology. Recently, it was discovered that both circulating, and tumor-associated, neutrophils possess functional plasticity when exposed to various inflammatory stimuli and in the tumor microenvironment. Neutrophils and their mediators can exert several pro-tumor activities in cancer and promote metastasis through different mechanisms. Angiogenesis plays a pivotal role in inflammation and tumor growth. Activated human neutrophils release several angiogenic factors [vascular endothelial growth factor-A (VEGF-A), angiopoietin-1 (ANGPT1), CXCL8, hepatocyte growth factor (HGF), and metalloproteinase 9 (MMP-9)] and form neutrophil extracellular traps (NETs). NETs promote tumor growth and metastasis formation through several mechanisms: they can awake dormant cancer cells, capture circulating tumor cells, coat and shield cancer cells, thus preventing CD8^+^- and natural killer (NK) cell-mediated cytotoxicity. ANGPTs released by endothelial and periendothelial mural cells induce platelet-activating factor (PAF) synthesis and neutrophil adhesion to endothelial cells. NETs can directly exert several proangiogenic activities in human endothelial cells and NETs induced by ANGPTs and PAF increase several aspects of angiogenesis in vitro and in vivo. A better understanding of the pathophysiological functions of NETs in cancer and angiogenesis could be of importance in the early diagnosis, prevention and treatment of tumors.

## 1. Introduction

Human neutrophils are conventionally considered fundamental players of the host response against a wide spectrum of different pathogens [1,2]. Peripheral blood neutrophils have the propensity to migrate into inflamed tissues in response to a plethora of chemotactic stimuli produced within the inflammatory site [3]. These cells kill pathogens through phagocytosis, the release of their potent antimicrobial arsenal, which includes cytoplasmic enzymes, oxidants (e.g., reactive oxygen species: ROS), and lipid mediators [4,5], and the formation of neutrophil extracellular traps (NETs) [6,7,8].

Increasing evidence implicates neutrophils in the pathogenesis of a broad spectrum of human disorders, including chronic inflammatory disorders, autoimmune diseases and cancer, in addition to infections [2,9]. Neutrophils are generated in the bone marrow and enter the bloodstream as terminally differentiated cells with a short lifespan. To maintain a stable number in the peripheral blood, neutrophils are produced at a rate of 10 × 10^11^–2 × 10^11^ cells per day in humans [10]. Neutrophil development and terminal differentiation are controlled by growth factors (e.g., GM-CSF, G-CSF), microRNAs, and other regulatory systems. Cytoplasmic granules of neutrophils are formed during the differentiation process from the promyelocyte stage onwards. Primary (or azurophilic) granules are formed in promyelocytes, secondary (or specific) granules in myelocytes, and tertiary (or gelatinase) granules in band cells [11].

Recent evidence indicates that “aged” peripheral blood neutrophils can return to the bone marrow, where they are phagocytosed by resident macrophages, which stimulate the release of “new” cells to maintain a stable number of peripheral blood neutrophils [12]. Neutrophil aging appears to be mainly regulated by the microbiome [13]. Neutrophils can also translocate from the interstitium to the intravascular space through a mechanism termed “reverse migration” [14,15].

Human neutrophils display several activating cell surface receptors that activate different intracellular signaling mechanisms to mediate effector functions [2]. Neutrophil-expressed G_i_ protein-coupled receptors include formyl peptide receptors recognizing bacterial products and mitochondria-derived danger signals [16], the LTB_4_ receptor BLT1 [17], chemokine receptors (e.g., CXCR1, CXCR2), and the C3a and C5a receptors sensing anaphylatoxins [18]. Immunoglobulins can activate the FcαRI and FcγRIIA receptors [19]. Lipopolysaccharide activates the TLR4 on human neutrophils [20,21], which also express various receptors for a constellation of cytokines, such as G-CSF, GM-CSF, TNF-α, IL-1, IL-4, IL-6, IL-13, and interferons [22,23,24,25,26].

In this review, we provide a general overview of the pathogenic roles of neutrophils in cancer with emphasis on NETs and angiogenesis.

## 2. Neutrophils and Cancer

Approximately 50% of the world’s population is affected by cancer at some point during their lifetime. Until few years ago, neutrophils received only limited attention in tumor immunology because they were considered short-lived cells with a circulating half-life of ~10 h in humans [9] and had limited transcriptional activity. However, in vivo labeling of human neutrophils has suggested that these cells survive in circulation for more than five days [27]. Moreover, there is evidence that cytokines secreted by tumor and immune cells, such as G-CSF, IL-1β, IL-6 or TNF-α can extend their lifespan [28]. At present, our understanding of the in vivo multiple functions of neutrophils in cancer patients remains limited.

Several investigators have reported that patients with advanced-stage cancers can have high counts of peripheral blood neutrophils [29], and the neutrophil-to-lymphocyte ratio (NLR) has been proposed as a prognostic factor for survival in several tumors [30,31,32]. During the past decade, the phenotypes and functions of neutrophils in cancer patients have started to be investigated in more detail [9,33].

There is compelling evidence that tumor-associated neutrophils (TAN) and their mediators are involved in tumor growth and progression, angiogenesis, and metastasis formation [4,34,35,36]. A decade ago, it was demonstrated that mouse neutrophils could undergo polarization towards an anti-tumor N1 or a protumor N2 phenotype. N1 neutrophils exert a direct anti-tumor effect mediated by ROS production and antibody-dependent cell cytotoxicity (ADCC) [37,38]. There is some evidence that N1 cells can activate CD8^+^ T cells and dendritic cells (DCs) and perhaps present tumor antigens [37,39,40]. By comparison, N2 neutrophils can promote tumor development through the remodeling of the extracellular matrix, angiogenesis and the production of pro-tumorigenic cytokines and chemokines [33,37,41]. The conditions promoting N1 or N2 polarization in vivo are largely unknown. Whether the N1/N2 profile described in mouse models is applicable to humans remains largely unknown.

In 2015 it was discovered that both circulating, and tumor-associated, neutrophils (TANs) possess some functional plasticity and can undergo activation when exposed to various stimuli in the tumor microenvironment (TME) [42,43]. For instance, transforming growth factor-β (TGF-β) can promote a pro-tumor N2 phenotype, whereas interferon-β (IFN-β) or the inhibition of TGF-β induce an anti-tumor N1 phenotype [37,44]. At least two neutrophil subpopulations have been identified in the peripheral blood of both cancer patients and mouse models: mature normal-density neutrophils (NDNs) and low-density neutrophils (LDNs) [45,46,47,48,49]. Mature NDNs possess an N1-like phenotype and can kill tumor cells, whereas LDNs have immunosuppressive properties [46,47,48]. Recently, multiple neutrophil subsets have been described in the circulation of both mouse models [50,51] and cancer patients [47,50,51,52,53].

In colon cancer, a better prognosis is associated with high neutrophil counts [54] and in breast cancer models, depletion of neutrophils causes disease progression [55]. Macrophages activate neutrophils through IFNs to produce chemokines and cytokines [56]. Neutrophils can induce DNA damage in cancer cells by releasing ROS and ATP [57,58]. Neutrophils promote tumor growth and the formation of metastasis by increasing angiogenesis [20,35,59], cell motility, migration, and invasion [60]. In mouse models, neutrophils promote metastasis [61], whereas neutrophil depletion prevents metastasis [62]. Neutrophils can adhere to circulating tumor cells, favoring their proliferation and metastasis [63]. Moreover, neutrophils provide lipids to tumor cells, fueling their proliferation and metastatic activity [64]. In different experimental models, neutrophils can exert anti-tumor activities by directly killing tumor cells and activating αβ T cells and CD4^+^ and CD8^+^ T cells [4].

Neutrophil extracellular traps (NETs) promote metastasis by several mechanisms [65]. Neutrophils elastase (NE) and cathepsin G (CG) [61] awaken dormant cancer cells by cleaving the extracellular matrix (ECM) protein lamin, generating an epitope that binds to tumor integrins, leading to the proliferation and migration of cancer cells [66,67]. NETs can also shield tumor cells blocking the activity of cytolytic T lymphocytes (CTLs) [68]. NETs can promote thrombosis in cancer patients [69] and a NET circulating biomarker [i.e., citrullinated histone 3 (H3cit)] is prognostic for venous thrombosis [70]. Collectively, these results indicate that the detection and inhibition of NET formation could have relevance in cancer diagnosis, prognosis, and metastasis prevention. Table 1 summarizes the main protumorigenic mechanisms of NETs in cancer.

## 3. Neutrophils, Angiogenesis and Lymphangiogenesis

Angiogenesis, the formation of new blood vessels from pre-existing ones [101], can represent a physiological or pathological process. Physiological angiogenesis is essential in embryonic development, tissue repair, the menstrual cycle and the growth of collateral circulation [102,103]. Pathological angiogenesis occurs during inflammation and tumor growth [103]. Angiogenesis is stimulated by hypoxia, cell metabolic demands, and lack of nutrients. These metabolic events stimulate hypoxia inducible factor (HIF) and molecules, such as mTOR (mammalian target of rapamycin) [104,105], which play a central role in the production of proangiogenic factors [106]. Angiogenesis is a complex process that requires the coordinated expression of several pro- and anti-angiogenic factors and their receptors on blood endothelial cells (BECs). The process is activated by angiogenic factors, such as vascular endothelial growth factors (VEGFs), angiopoietins (ANGPTs), platelet-derived growth factor (PDGF), basic fibroblast growth factor (bFGF), epidermal growth factor (EGF), and CXCL8/IL-8. Several anti-angiogenic factors, such as angiostatin, endostatin, and angiopoietin 2 (ANGPT2) [107], and the VEGF-A_165b_ isoform [35] modulate inflammatory and tumor angiogenesis.

Vascular endothelial growth factors (VEGFs), including VEGF-A, VEGF-B, and placental growth factors (PlGF) are the most important angiogenic factors [108]. These factors exert their biological activities through the activation of two tyrosine kinase receptors, VEGFR1 and VEGFR2, on blood endothelial cells [109,110]. VEGF-C and VEGF-D promote lymphangiogenesis through the engagement of VEGFR3 on lymphatic endothelial cells [111,112]. Angiopoietins (ANGPTs) are ligands for the tyrosine kinase TIE2 receptor [113,114] and have a pivotal role in blood vessel homeostasis [103]. In humans, there are two known ANGPTs, namely, ANGPT1 and ANGPT2 [115]. ANGPT1, produced by perivascular mural cells, is an agonist, while ANGPT2, contained in Weibel–Palade bodies, is an antagonist of the TIE2 receptor on BECs [116]. ANGPT1 stabilizes the endothelial cell junction integrity and inhibits the increase in vascular permeability caused by VEGF-A. By contrast, ANGPT2 by competitive antagonism of TIE2 receptor inhibits this stabilization [117,118]. In normal conditions, ANGPT2 is minimally expressed but it increases at the site of inflammation and in tumors [119,120]. ANGPT2 levels are elevated in cancer, increasing angiogenesis, tumor growth, and metastasis formation [118].

ANGPT1 phosphorylates the TIE2 receptor leading to PI3K/AKT activation, which causes phosphorylation (inactivation) of the transcription factor FOXO1 (Forkhead box O). This process leads to vessel stabilization. ANGPT2 is an antagonist of the TIE2 receptor, which allows PI3K/AKT inactivation and prevents phosphorylation (inactivation) of FOXO1 [121]. This induces the release of ANGPT2 from BECs [122,123]. In pathological conditions, such as cancer, AKT inactivation, caused by weak ANGPT1-TIE2 signaling, results in the activation of Foxo1 and the expression of ANGPT2. In this condition, ANGPT2 promotes TIE2 phosphorylation to compensate for the ANGPT1-induced activation of TIE2, leading to chemotaxis of endothelial cells and tube formation.

Human neutrophils produce and release a wide spectrum of proangiogenic factors and play key roles in different models of inflammatory and tumor angiogenesis [124,125,126]. VEGF-A, the most potent proangiogenic molecule, is present in human neutrophils [35,83] and can be released in response to *N*-formyl-methionyl-leucyl-phenylamine (fMLF) [35,125], TNF-α [84,127], lipopolysaccharide (LPS) [20,35,127], G-CSF [128], and PMA [35,125]. We have shown that highly purified human neutrophils contain preformed VEGF-A ~ ANGPT1 > VEGF-B [35]. Human neutrophils constitutively express *VEGF-A*, *VEGF-B*, and *ANGPT1* mRNAs. We also found that several human recombinant phospholipases A_2_ (sPLA_2_) (group V and group X) induced the release of ANGPT1 > VEGF-A > CXCL8 from neutrophils [35]. These results were paralleled by the release of β-glucuronidase, a marker of exocytosis. By contrast, no secretion of the angiogenic factor VEGF-B could be observed in these experiments. The release of proangiogenic factors from human neutrophils was inhibited by the preincubation of cells with brefeldin A (inhibitor of cellular transport and protein secretion) or cycloheximide (inhibitor of protein synthesis). These results are compatible with the hypothesis that sPLA_2_ can induce the de novo synthesis of several proangiogenic factors from human neutrophils.

Angiogenesis is the result of a dynamic balance between proangiogenic and anti-angiogenic factors. VEGF-A exists in multiple isoforms possessing strikingly contrasting properties [129]. VEGF-A_165_ is the first VEGF-A isoform described [130], and its expression, signaling, and roles in inflammatory and tumor angiogenesis, have been widely investigated [20,35,101,131]. Other isoforms (i.e., VEGF-A_121_, VEGF-A_145_, VEGF-A_148_, VEGF-A_183_, VEGF-A_189_, and VEGF-A_206_) are generated by alternative splicing of exon 6 and 7 [129]. VEGF-A_165b_ is an additional isoform generated by exon 8 distal splice site selection. Thus, *VEGF-A* mRNA splicing generates two families of proteins that differ by their carboxy-terminal six amino acids [129,132]. These conformational changes result in the inability of VEGF-A_165b_ to bind neuropilin 1 (NRP1), a co-receptor of VEGFR2 [133]. As a result, VEGF-A_165b_ fails to induce VEGFR2 tyrosine phosphorylation and activates the downstream signaling pathway that characterizes the proangiogenic isoform VEGF-A_165a_ [133,134]. The translational relevance of these observations is supported by the involvement of VEGF-A_165b_ and its anti-angiogenic activities in different human disorders [135,136,137].

We found that highly purified human neutrophils constitutively express *VEGF-A_165b_* mRNA [35]. Interestingly, human recombinant sPLA_2_ group V, but not group X, caused VEGF-A_165b_ release from neutrophils. These results indicate that sPLA_2_ group V selectively modulates the release of anti-angiogenic factors from human neutrophils. Further studies are needed to verify whether other immunological stimuli can induce the release of anti-angiogenic factors (e.g., VEGF-A_165b_) from human neutrophils.

More recently, we have extended the previous observation showing that untreated neutrophils contain hepatocyte growth factor (HGF) > VEGF-A ~ CXCL8 [20] and that LPS and fMLF induced the release of these angiogenic factors. Matrix metalloproteinase 9 (MMP-9) is also secreted by human neutrophils and plays an important role in promoting angiogenesis by counteracting the effects of anti-angiogenic molecules and by inducing the release of VEGF-A in TME [138,139].

Lymphangiogenesis, the formation of new lymphatic vessels from pre-existing ones, is limited in healthy adults but occurs during pathological conditions, such as inflammation, tissue repair, and tumor growth [140]. VEGF-C and VEGF-D are key regulators of lymphangiogenesis by activation of the VEGFR3 receptor on lymphatic endothelial cells (LECs) [141]. Previous studies have demonstrated that tissue-resident immune cells, such as human macrophages [142,143] and mast cells [110,112,144], are a major source of lymphangiogenic factors. When we investigated the effects of sPLA_2_ on the release of lymphangiogenic factors from human neutrophils, we found that these cells do not express *VEGF-C/VEGF-D* mRNAs. Moreover, several sPLA_2_ do not cause the secretion of these lymphangiogenic factors from neutrophils [35]. Further studies should investigate whether other immunological stimuli can induce the expression and release of lymphangiogenic factors from these cells.

## 4. NET Formation

Neutrophil extracellular traps (NETs) are pleiotropic networks composed of a DNA scaffold associated with several granule proteins and released by activated neutrophils in a process commonly termed NETosis [6,145]. NETs can trap several bacteria [6,146,147,148,149], fungi [150,151], and viruses [152,153], enabling their subsequent clearance [7,8,154].

A pioneering study described a novel form of neutrophil death induced by the prolonged incubation (3 h) with phorbol 12-myristate 13-acetate (PMA), which differed from apoptosis and necrosis [155]. PMA induced the loss of chromatin compactness, the rupture of the nuclear and cytoplasmic membranes, and the release of nuclear DNA. This process was ROS-dependent and led to neutrophil cell death [155]. Subsequently, Brinkmann and collaborators elegantly described NET composition and their antimicrobial properties [6]. Different immunological stimuli [IL-8/CXCL8, IFN-α/IFN-γ/C5a, GM-CSF/C5a, and lipopolysaccharide (LPS)] induce NET formation from human neutrophils within 2–4 h after neutrophil activation [6,8,145,146,156]. In addition, several bacteria [6], fungi [157], viruses [158], anti-neutrophil cytoplasmic antibodies [159,160] and calcium ionophores [161,162] can also induce NET formation. The latter form of NET release requires cell death [161,163,164,165,166] and can be defined as “suicidal” NETosis [73]. PMA is a classical stimulus inducing suicidal NETosis [6,155]. In this process, PMA activates protein kinase C (PKC) causing calcium release from intracellular stores [167], ROS production and p38 MAPK activation [164]. Myeloperoxidase (MPO) and neutrophil elastase (NE) are released from the azurophilic granules [168] and translocate into the nucleus [169]. Activated peptidyl-arginine deiminase 4 (PAD4) translocates to the nucleus and catalyzes the deamination of histones H2A, H3 and H4, converting the arginine residues into citrulline. The loss of positive charge of histones results in a marked decrease of chromatin compactness [170,171]. Nuclear and mitochondrial membranes disintegrate and the intracellular content is released in the extracellular space and the neutrophil dies [172].

Several groups of investigators demonstrated the existence of a “vital” form of NET formation, in which the intracellular content of neutrophils is released in the extracellular space [8,73,146,147,173,174,175]. Vital NET formation rapidly occurs (5–60 min) after neutrophil activation and can be associated with vesicle release filled with mitochondrial DNA [8,73]. This process occurs via a rapid mechanism [147] and neutrophils are still alive to exert different functions [173,176,177]. This NET formation is cytoskeleton and glycolysis dependent but it occurs independently from oxidant production [8,146,174,175]. We have demonstrated that conditioned media from anaplastic thyroid cancer (ATC) cell lines induced vital release of NETs containing mitochondrial DNA [73].

## 5. NETs in Cancer

In addition to their role in host defense, NETs play a crucial role in several inflammatory disorders, such as autoimmune [178,179,180,181] and allergic diseases [182,183,184,185,186,187,188,189], pulmonary [112,190,191], cardiovascular [192,193,194], and autoinflammatory disorders [49], and sepsis [195]. NETs are also involved in venous and artherial thrombosis [196].

Several clinical and experimental studies emphasize the association among NET formation and tumor development, the formation of metastasis, and cancer-associated thrombosis [71,74,93,197,198,199,200,201]. Several reviews have recapitulated the roles of NETs in cancer [174,202,203,204,205,206,207,208].

NETs can stimulate endothelial-to-mesenchymal transition (EMT) [71], which is involved in tumorigenesis [209]. In murine models, NETs facilitate experimental cancer development [75,93,210,211]. NETs released from LDNs promote intestinal tumorigenesis [212]. In a murine model, genetic deletion of PAD4 reduces NETs, decreases tumor development and improves survival [76]. NETs accumulate in TME and promote experimental melanoma growth [81]. Incubation of NETs with melanoma cells reduces their migration and viability [213]. Circulating and intratumoral NETs are associated with an unfavorable prognosis in human B cell lymphomas [77]. High circulating NET biomarkers are correlated with a worse prognosis in metastatic colorectal cancer patients [201]. IL-8/CXCL8 induces NET formation and is associated with colorectal cancer liver metastasis [78]. IL-8/CXCL8, produced by a variety of human cancer cells [82,214,215], causes NET formation from neutrophils and granulocytic myeloid-derived suppressor cells (PMN-MDSCs) [73,77,192,216,217]. Several chemokines/cytokines (CXCR1/CXCR2 agonists, G-CSF, TGF-β) released in TME induce NET formation from human and murine neutrophils [16,68,216,218,219].

The adhesive properties of NETs favor their binding to pathogens and cancer cells [220]. NETs can trap circulating tumor cells (CTCs), thus promoting metastasis [221,222]. In an in vivo model, the dismantling of NETs by DNases inhibited NET trapping of CTC and the formation of metastasis [74]. Intravenous administration of a metastatic cell line favored the deposition of NET in the lungs and the formation of the metastatic niche [75]. MPO, a product of neutrophils, and H3Cit, a biomarker of NETs, were present in primary breast cancer and liver metastasis [79]. NET DNA induces cancer cell chemotaxis through the interaction with a membrane protein on tumor cells, promoting metastasis formation. NETs can promote metastasis through endothelial cell (EC) damage [223]. In fact, NET-associated NE increased EC permeability through the proteolysis of vascular endothelial (VE)-cadherin [71].

Due to poor angiogenic activity and the protective role of immune cells, metastatic cancer cells can remain dormant for an extended period [224,225]. Lipopolysaccharide (LPS) administration to mice bearing dormant cancer cells (DCCs) in the lung causes local NET formation [66], whereas neutrophil depletion blocks LPS-induced awakening of DCCs. In this study, NET formation and awakening of DCCs were inhibited by PAD4 inhibition or digestion of DNA. Collectively, these results suggest that NETs formed by inflammatory stimuli can induce the awakening of DCCs in different mouse models.

**Figure 1:**
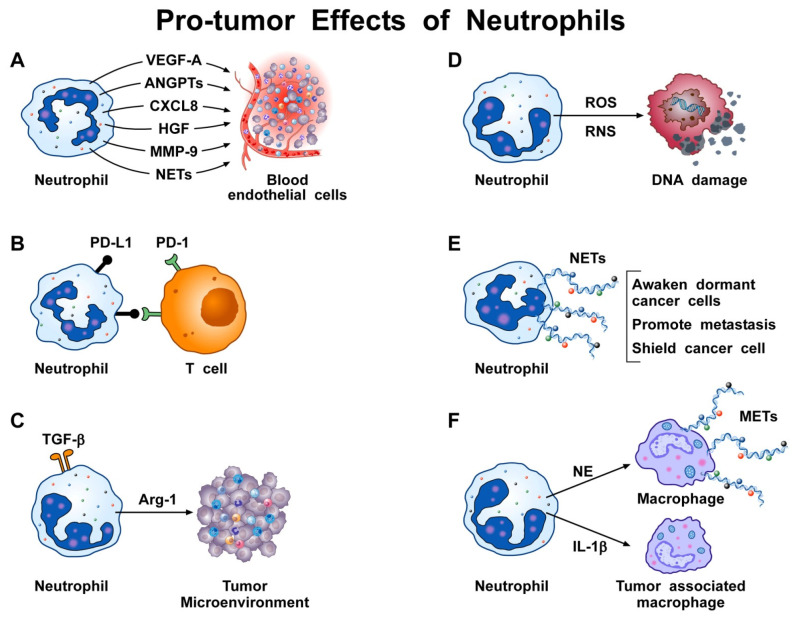
Neutrophils can exert several pro-tumor activities in cancer. (**A**) Activated human neutrophils release VEGF-A [35,84,88,127,128], ANGPTs, Bv8 protein [226], CXCL8 [20,35], hepatocyte growth factor (HGF) [87], matrix metallopeptidase 9 (MMP-9) [138], and NETs that promote angiogenesis. (**B**) Human neutrophils can express ligands of immune checkpoints such as PD-L1. The interaction between PD-L1 and PD-1 on T cells inhibits the anti-tumor immune response of T cells. (**C**) TGF-α activates a surface receptor on neutrophils [227] inducing the release of arginase 1 (Arg-1), which causes T cell dysfunction and suppression of T cell-mediated anti-tumor immune response [37,228]. (**D**) Human neutrophils produce reactive oxygen species (ROS) and reactive nitrogen species (RNS) which cause DNA damage and genetic instability in epithelial cells [57,58,229]. (**E**) Neutrophil extracellular traps (NETs) awaken dormant cancer cells (DCCs) [66], promote metastasis formation [71,223], and shield cancer cells, thus impairing the cytotoxicity mediated by CD8^+^ T cells and NK cells [68]. (**F**) Activated neutrophils release neutrophil elastase (NE), which triggers the release of macrophage extracellular traps (METs) [230] and produces several proinflammatory cytokines (e.g., IL-1β) that activate tumor-associated macrophages (TAMs).

Chemokines activating the CXCR1 and CXCR2 receptors, produced by melanoma and colon carcinoma cell lines, induce NET formation which shields cancer cells against NK- and T cell-mediated cytotoxicity [68]. Reparixin (CXCR1 and CXCR2 antagonist) or a mAb blocking CXCR1 inhibits the in vivo formation of NETs in tumors. Incubation of cytotoxic lymphocytes (CTL) or NK cells with activated neutrophils induces NET release, which impairs cytotoxicity by shielding cancer cells. In vivo experiments by intravital microscopy showed that NETs impaired cytotoxic cell contact with tumor cells. Collectively, these results indicate that coating of cancer cells by NETs prevents cytotoxicity induced by CD8^+^T cells and NK cells [68]. Figure 1 schematically summarizes some of the pro-tumor activities of neutrophils.

NETs found in venous thromboembolism (VTE) and arterial thromboembolism (ATE) [231,232,233,234] can be linked to a pro-thrombotic state in cancer [69,93,94]. Pancreatic cancer, typically associated with VTE [235,236,237], has a poor prognosis [238,239]. NET biomarkers are augmented in hepatocellular carcinoma-related thrombosis [240], in tumor-associated stroke [241], and myeloproliferative neoplasms [242] and their increase predicts the risk of VTE in cancer patients [70]. It is important to note that quantitative analysis of DNA as a surrogate of NET formation can be misleading because increased circulating levels of DNA complexes can result from any cell death associated or not with neutrophilic inflammation [243].

NETs are now considered a promising therapeutic target in cancer [208]. It is well established that NETs amplify the metastatic potential of cancer cells [79,221,223]. Therefore, the inhibition of their formation, or the promotion of their resolution, have been proposed as therapeutic strategies in several experimental tumors [67,68,75]. DNase, degrading chromatin within the NETs, is one of the promising strategies to interfere with NET formation and activity [179]. DNase treatment reduces disease burden and metastasis in murine models of breast [244] and lung cancers [74], respectively.

## 6. NETs Modulate Inflammation

Increasing evidence demonstrates that NETs can promote inflammation by inducing proinflammatory cytokines from different immune cells [245,246]. In particular, histones, the major citrullinated proteins in NETs, activate the transcription of IL-1β in mononuclear cells by binding and activating TLR4. Moreover, NET DNA synergizes with citrullinated histones to induce IL-1β by promoting intracellular TLR4 translocation to endosomes [245]. In addition, NETs isolated from activated human neutrophils resulted in the activation of several cellular functions [246]. In particular, NETs induced the secretion of CXCL8/IL-8, but not of TNF-α, by human neutrophils.

A recent study demonstrated that microRNAs (miRNAs) can be associated with NET scaffolds [247]. Human neutrophils activated by different stimuli formed NETs carrying miRNA cargo. Interestingly, NET-associated miRNA-142-3p can be transferred to macrophages leading to a reduced expression of protein kinase Cα (PKCα), which modulates TNF-α production [247]. Therefore, the presence of mi RNA-142-3p in NETs regulating the production of inflammatory cytokines could be a negative feedback loop tuning the inflammatory reaction. It will be interesting to verify whether other miRNAs present in NET supernatants can play a pro-inflammatory or anti-inflammatory role. Collectively, these recent studies highlight two novel mechanisms through which NET-associated molecules could propagate or modulate inflammation.

## 7. NETs and Angiogenesis

Angiogenesis occurs in several inflammatory diseases [112,120,144] and tumors [73,248]. VEGFs, including VEGF-A, VEGF-B, and PlGF are the major angiogenic factors [21]. The angiopoietins (ANGPTs) are also important angiogenic factors [113,119,249]. Peri-vascular mural cells (pericytes) [250] and certain immune cells [251,252] produce ANGPT1, an agonist of the TIE2 receptor on ECs [253,254]. ANGPT2, stored in Weibel–Palade bodies, is an antagonist of the TIE2 receptor [116,255]. ANGPT1 and ANGPT2 exert several proinflammatory and proangiogenic activities on ECs and leukocytes in vitro and in vivo [256,257,258]. Both ANGPT1 and ANGPT2 can induce endothelial platelet-activating factor (PAF) synthesis and neutrophil adhesion into ECs [256,257,258,259].

Aldabbous and collaborators demonstrated that NETs directly promote angiogenesis in vitro and in vivo [85]. NETs generated by prolonged incubation of human neutrophils with PMA induce pro-angiogenic responses in a classical matrigel tube formation assay and a 3-dimensional spheroid sprouting assay. NETs induce angiogenesis (increased tube length, number of sprouts, and sprouting area) in human pulmonary artery endothelial cells (HPAECs). To investigate the effect of NETs on angiogenesis in vivo, they found that NET DNA injected subcutaneously into mice increases vascularization [85].

Sirois and colleagues have found that both ANGPT1 and ANGPT2 induce PAF synthesis in human neutrophils [256]. However, only ANGPT1, but not ANGPT2, induces cytokine release (e.g., IL-1β, IL-8/CXCL8) from human neutrophils [260,261]. Recently, the same group found that prolonged incubation (3 h) of human neutrophils with ANGPT1 and ANGPT2, alone or in combination, increases NET formation [86]. The release of NETs is mediated by the activation of TIE2 and requires the production of ROS. A PAD4 inhibitor, GSK484, completely inhibits ANGPT-induced NET formation. In this experimental model, a PAF receptor antagonist inhibited pro-angiogenic activity of NETs is also assessed using a classical matrigel assay of angiogenesis. NETs induced by ANGPT1/2 or PMA increase capillary-like tube length, the number of loops and tubule area [86]. Collectively, these studies demonstrate that ANGPTs can promote the release of NET, which exerts proangiogenic activities in vivo and in vitro. Table 2 summarizes the main proangiogenic mechanisms of NETs.

Macrophages, important resident immune cells in TME [143,262], are critical sentinels in tumor immunity, modulating angiogenesis and lymphangiogenesis [21,263] and surveilling against tumors [264,265]. Recent studies have demonstrated that activated macrophages can release extracellular DNA traps, also called macrophage extracellular traps (METs) [95,230,266]. There is some evidence that METs and TAMs contribute to cancer progression [267]. Aside from the interactions between several microorganisms and macrophages [268], other factors can induce MET formation. Neutrophil elastase, a major component of azurophilic granules of human neutrophils [168], can be released in association with NETs. Recently, it has been demonstrated that NE triggers the release of METs from human and mouse macrophages [230]. This observation highlights a novel interaction between human neutrophils and macrophages, two important immune cells that play significant roles in various aspects of tumorigenesis.

Mast cells [95,269,270,271,272,273,274], eosinophils [149,150,183,275,276,277,278], basophils [186,275,279], and macrophages [95,230], which are present in TME [143,144,208,248,280,281], can also release extracellular DNA traps. Further studies are needed to evaluate whether immunological stimuli (e.g., ANGPTs) can induce the formation of extracellular DNA traps from human mast cells, macrophages, eosinophils, and basophils.

## 8. Conclusions

There is now compelling evidence that neutrophils and NETs can promote several stages of tumorigenesis and metastasis formation. However, there is some evidence that neutrophils and NETs also exert reparative effects in the context of tumor inflammation [198,213]. Moreover, recent studies indicate that different components of NETs can propagate [245,246] or modulate inflammation [247]. Such dichotomy in the outcome of neutrophil activity is fascinating and intriguing [282]. It is presently unclear whether different activities of neutrophils depend on the opposite functions of their subsets or if environmental signals influence their plasticity.

There is compelling evidence that human neutrophils constitutively express [20,35,83,138,139] and release various proangiogenic molecules. Recently, we have found that human neutrophils also express and release the anti-angiogenic VEGF-A_165b_ [35]. NETs can exert proangiogenic effects in vivo and in vitro [85,86,260,261]. Future studies should investigate whether human neutrophils activated by different stimuli can form NETs carrying proinflammatory and anti-inflammatory molecules.

The ability of NETs to trap a plethora of microorganisms has generated much attention [1,2], but it is their pathogenic potential in cancer that is attracting increasing enthusiasm [64,66,68,70,208,223]. NETs seem to play multiple roles in tumor growth, angiogenesis and metastasis formation. Given the multitude of NET-associated proteins, novel NET functions are likely to emerge. A better understanding of the pathophysiological functions of NETs in cancer and angiogenesis could be of paramount importance in the early diagnosis of tumors, in the prevention of metastasis, and when designing novel strategies for cancer treatment.

## Figures and Tables

**Table 1:** Main protumorigenic mechanisms of NETs in cancer.

Mechanisms	References
NETs drive endothelial-to-mesenchymal transition	[71,72]
NETs promote experimental tumor growth	[71,73,74,75,76,77,78,79,80]
NETs promote human tumor growth	[73,74,75,77,78,80,81,82]
NETs promote angiogenesis	[35,41,83,84,85,86,87,88]
NETs trap circulating cancer cells	[68,74,89]
NETs awaken dormant cancer cells	[66]
NETs promote metastasis formation	[74,79,90,91,92]
NETs shield cancer cells from cytotoxicity	[68]
NETs promote cancer-associated thrombosis	[93,94,95,96,97,98,99,100]

**Table 2:** Main proangiogenic mechanisms of NETs.

Mechanisms	References
NETs induce increased capillary tube length, number of sprouts, and sprouting area of endothelial cells	[85]
Angiopoietin 1 (ANGPT1) and angiopoietin 2 (ANGPT2), alone or combined, induce NET formation	[86]
ANGPT-mediated NETs increase human endothelial cell tube length and the number of loops	[86]
Human neutrophils sustain angiogenesis through the release of VEGF-A, HGF, BV8, and MMP9	[35,41,83,84,87,88]

## Data Availability

Not applicable.

## Abbreviations

ANGPT	angiopoietin
ANGPT1	angiopoietin 1
ANGPT2	angiopoietin 2
Arg-1	arginase 1
ATC	anaplastic thyroid cancer
ATE	arterial thromboembolism
BEC	blood endothelial cell
bFGF	basic fibroblast growth factor
CTL	cytolytic T lymphocyte
DC	dendritic cell
EC	endothelial cell
EGF	epidermal growth factor
EMT	endothelial-to-mesenchymal transition
FOXO1	Forkhead box O
H3cit	citrullinated histone 3
HGF	hepatocyte growth factor
HIF	hypoxia inducible factor
HPAEC	human pulmonary artery endothelial cells
LDN	low-density neutrophil
LEC	lymphatic endothelial cell
LPS	lipopolysaccharide
mAb	monoclonal antibody
MET	macrophage extracellular trap
MMP-9	metalloproteinase 9
MPO	Myeloperoxidase
NDN	normal-density neutrophil
NE	neutrophil elastase
NET	neutrophil extracellular trap
NK cell	natural killer cell
NLR	neutrophil-to-lymphocyte ratio
NRP1	neuropilin 1
PAD4	peptidyl-arginine deiminase 4
PAF	platelet-activating factor
PKC	protein kinase C
PlGF	placental growth factor
PMN-MDSC	neutrophils and granulocytic myeloid derived suppressor cell
sPLA_2_	secreted phospholipases A_2_
TAM	tumor associated macrophage
TME	tumor microenvironment
TNF-α	tumor necrosis factor-α
VEGF	vascular endothelial growth factor
VEGFR	vascular endothelial growth factor receptor
VTE	venous thromboembolism
